# Rapid detection of SARS-CoV-2 with CRISPR-Cas12a

**DOI:** 10.1371/journal.pbio.3000978

**Published:** 2020-12-15

**Authors:** Dan Xiong, Wenjun Dai, Jiaojiao Gong, Guande Li, Nansong Liu, Wei Wu, Jiaqiang Pan, Chen Chen, Yingzhen Jiao, Huina Deng, Junwei Ye, Xuanxuan Zhang, Huiling Huang, Qianyun Li, Liang Xue, Xiuming Zhang, Guanghui Tang

**Affiliations:** 1 Yaneng Biotech Co. Ltd., Fosun Pharma, Shenzhen, China; 2 Medical Laboratory of Shenzhen Luohu People’s Hospital, Shenzhen, China; 3 Singuway Biotech Co. Ltd., Shenzhen, China; 4 Department of Neurology, Hwa Mei Hospital, University of Chinese Academy of Sciences, Ningbo, China; University of Wisconsin-Madison, UNITED STATES

## Abstract

The recent outbreak of betacoronavirus Severe Acute Respiratory Syndrome Coronavirus 2 (SARS-CoV-2), which is responsible for the Coronavirus Disease 2019 (COVID-19) global pandemic, has created great challenges in viral diagnosis. The existing methods for nucleic acid detection are of high sensitivity and specificity, but the need for complex sample manipulation and expensive machinery slow down the disease detection. Thus, there is an urgent demand to develop a rapid, inexpensive, and sensitive diagnostic test to aid point-of-care viral detection for disease monitoring. In this study, we developed a clustered regularly interspaced short palindromic repeats (CRISPR)-CRISPR associated proteins (Cas) 12a-based diagnostic method that allows the results to be visualized by the naked eye. We also introduced a rapid sample processing method, and when combined with recombinase polymerase amplification (RPA), the sample to result can be achieved in 50 minutes with high sensitivity (1–10 copies per reaction). This accurate and portable detection method holds a great potential for COVID-19 control, especially in areas where specialized equipment is not available.

## Abstract 2

The outbreak of SARS-CoV-2 has caused great damage to human society, and early detection of this virus is important to contain its spread and save lives. Existing diagnostic methods are complex and require well-trained personnel, but this study describes a fast and accurate method which can be operated by lay-users and whose results can be visualized by the naked eye.

## Introduction

Severe Acute Respiratory Syndrome Coronavirus 2 (SARS-CoV-2), which emerged in the city of Wuhan, China, has become a serious public health concern [1]. This virus spread rapidly, with over 30 million reported cases and 946,000 deaths worldwide as of September 18, 2020[2,3]. As numerous infections are asymptomatic, the total number of infections remains unknown [4]. Thus, there is an increasing demand for a rapid and sensitive screening method to differentiate infected from healthy individuals.

The existing nucleic acid detection methods, such as quantitative reverse transcription polymerase chain reaction (RT-qPCR), are sensitive and accurate, but inadequate access to reagents and machinery has limited their application for disease control. For instance, RT-qPCR requires highly purified samples and long reaction times (normally more than 2 hours) as well as well-trained personnel. These limitations may hinder the prescription and administration of antiviral agents to patients.

The clustered regularly interspaced short palindromic repeats (CRISPR) and Cas (CRISPR associated proteins) are adaptive immune systems in archaea and bacteria, which recognizes and degrades foreign nucleic acid under the guidance of a sequence-specific RNA molecule [5–8]. Some Cas enzymes (including Cas12a, Cas12b, Cas13a, Cas13b, and Cas14) display collateral cleavage activity after binding to their specific targets, which has been fully evaluated for diagnostic use to detect nucleic acids[5,9–12]. Upon target recognition, the activated Cas nucleases indiscriminately cleave nearby single-stranded DNA (ssDNA). By introducing an ssDNA reporter labeled with a fluorophore and quencher or alternatively with a fluorophore and biotin into an in vitro reaction, the cleavage could be detected with a fluorescent reader or a lateral flow paper strip.

Although all of these proteins are RNA-guided nucleases, Cas13a and Cas13b are RNases, while Cas12a, Cas12b and Cas14 are DNases [5,8,11,13,14]. Therefore, the Cas13a- and Cas13b-based detections require a transcription step by RNA polymerase and, in contrast, Cas12a, Cas12b, and Cas14 can directly use DNA as substrates. The pre-amplification of nucleic acid with an isothermal or PCR method is normally required to enhance the detection sensitivity [5,14,15]. By combining with the recombinase polymerase amplification (RPA) technology, Cas12a and Cas13a have both been shown to permit single-molecule detection in a given reaction [5,9]. Additionally, a class of diagnostic methods using Cas9 or dCas9 that lacks collateral effect have also been reported for nucleic acid detection [16,17]. These paradigms illustrate the potential of CRISPR-Cas systems as in-site diagnostic tools for the detection of SARS-CoV-2 and, indeed, several CRISPR-based tests have been recently developed [18–21].

**Fig 1:**
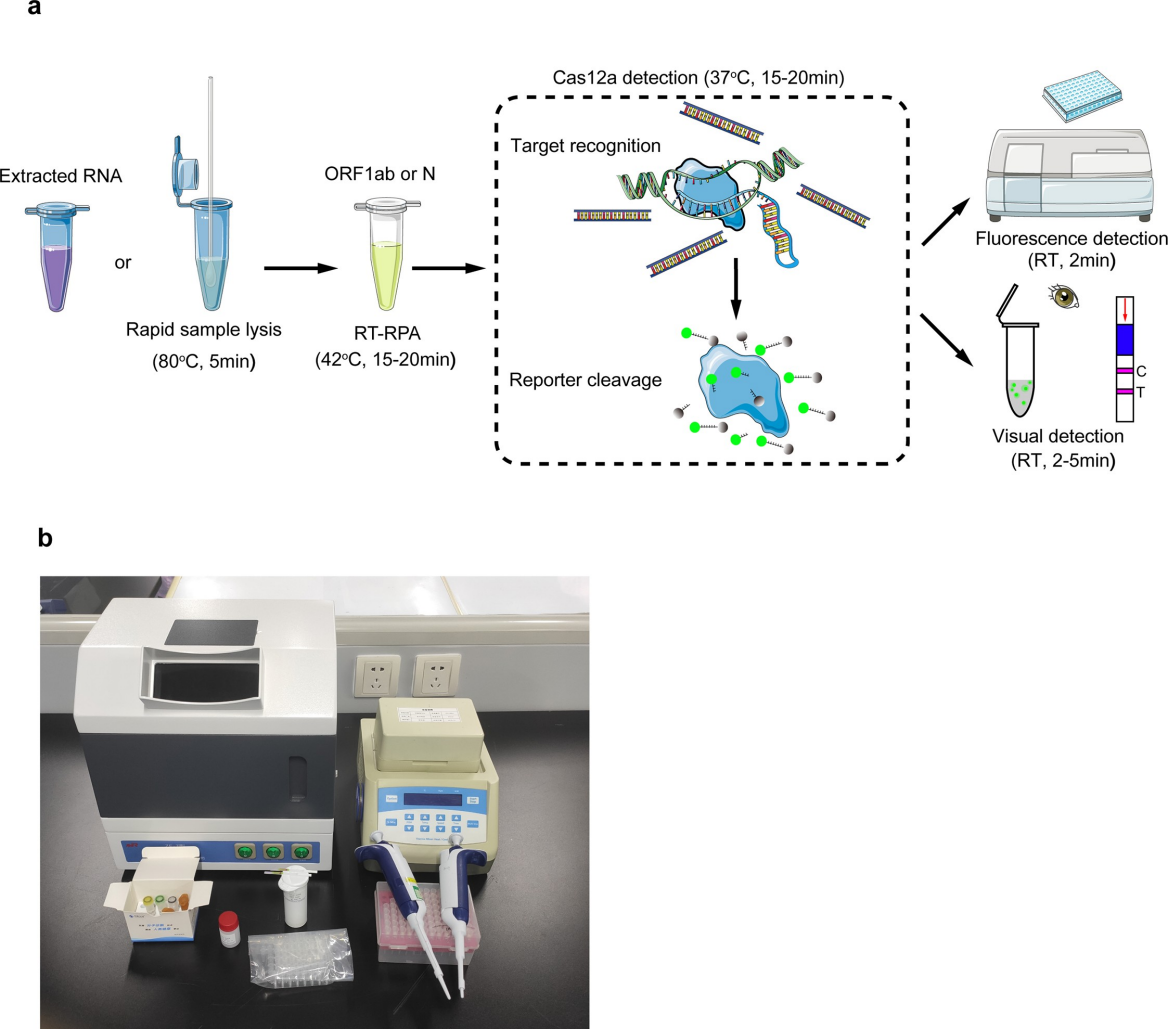
Workflow of Cas12a-based detection for SARS-CoV-2. Extracted RNA or lysed samples can be used as an input for RPA-Cas12a-based detection. (**a**) The results can be achieved within 50 minutes using a fluorescent reader or with visual detection methods. (**b**) The minimum equipment needed to run the assay following RNA extraction or rapid sample lysis includes UV light imagers, heat blocks (42°C and 37°C), pipettes and tips, Eppendorf tubes, reagents, and lateral flow strips. Cas, CRISPR associated proteins; RPA, recombinase polymerase amplification; RT, room temperature; SARS-CoV-2, Severe Acute Respiratory Syndrome Coronavirus 2; UV, ultraviolet.

In this study, we present an approach for the visual detection of the ORF1ab and N genes of SARS-CoV-2 using CRISPR-Cas12a system. By combination of a sample processing method and the RPA technology with Cas12a-based detection, the sample to result can be obtained in 50 minutes with a fluorescent reader or via the naked eye (Fig **1**A). Because our assay needs minimal equipment for a visual detection of SARS-CoV-2 (Fig **1**B), its application may help the control of this pandemic.

## Results and discussion

**Fig 2:**
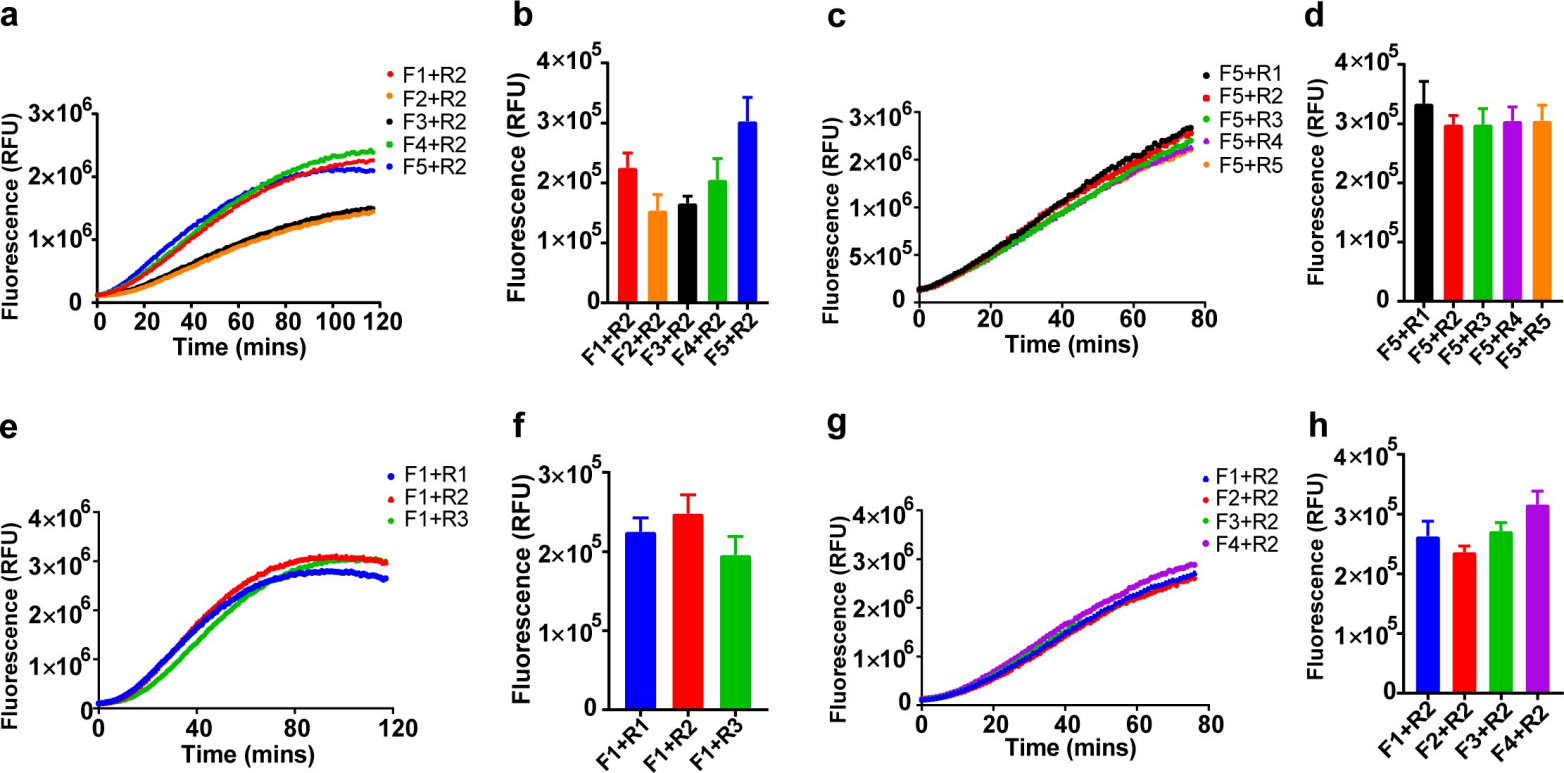
Primer screening. Primers for ORF1ab (**a–d**) and N (**e–h**) were screened using a single forward or reverse primer against the corresponding reverse or forward primers, selecting the best performing primer and then using it to perform second round screening to determine the primer pairs with the best performance. Fluorescent signal was obtained at 10 minutes for the Cas12a reaction. The data are presented as the means ± SD (*n* = 3; **b**, **d**, **f**, **h**). Numerical source data underlying this figure can be found in S1 Data. Cas, CRISPR associated proteins; RFU, relative fluorescence unit; SD, standard deviation.

As the sequences of oligonucleotides are crucial to a rapid and sensitive RPA, primers need to be screened to establish a preferred detection method. We tested 18 and 15 primer combinations for the target genes of ORF1ab and N, respectively, using 100 copies of RNA for each reaction. To screen out the best primer set for the ORF1ab gene, a reverse primer (R2) was used to against forward primers (F1 to F5), selecting the best forward primer (F5) and then using it to screen all the reverse primers (R1 to R5). The primer pair with the best performance was identified and used in subsequent experiments (F5+R1, Fig 2A–2D). Primers for the N gene of SARS-CoV-2 were subjected to the same procedure, and the primer set of F4+R2 exhibited the best performance (Fig 2E–2H).

**Fig 3:**
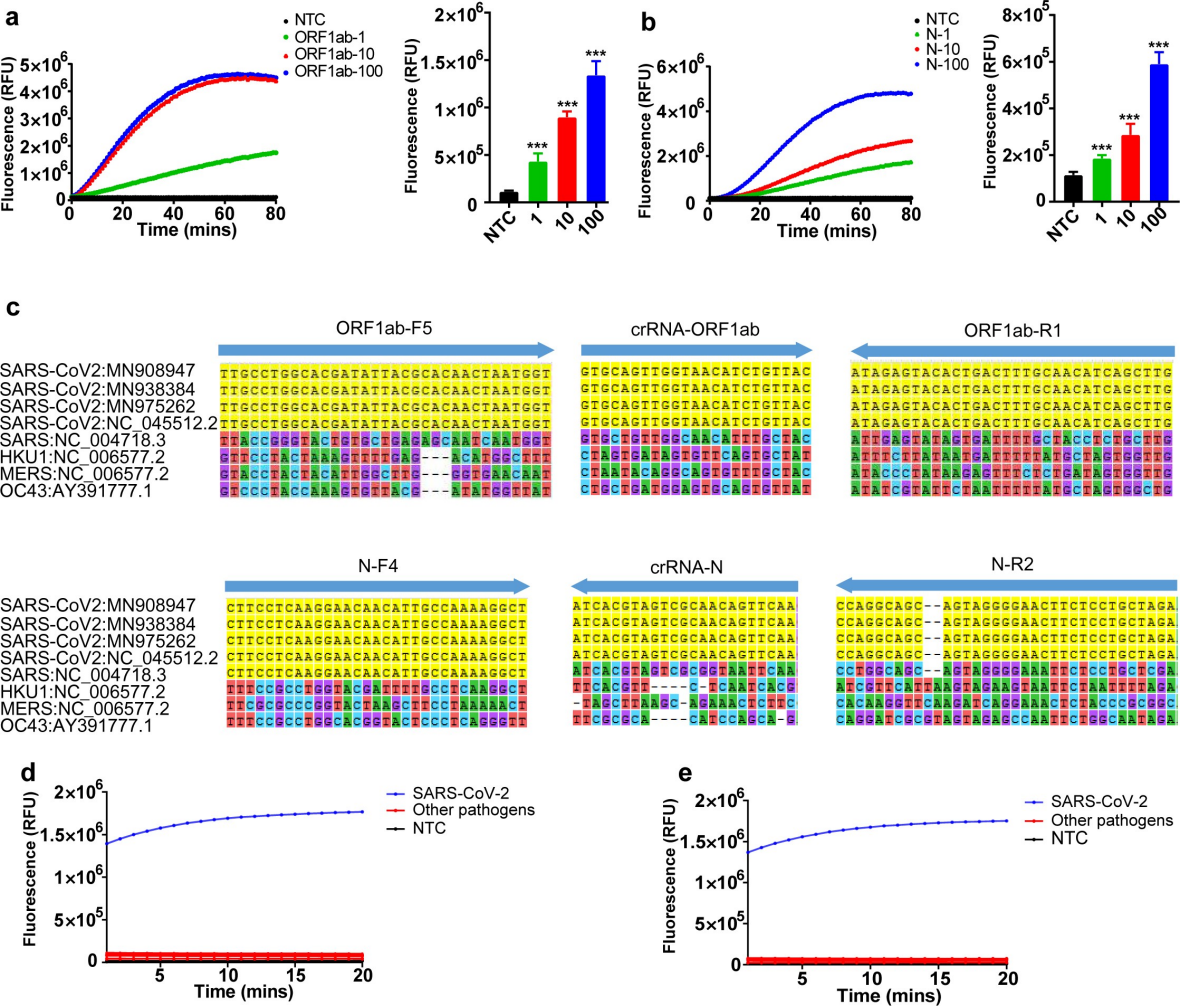
Sensitivity and specificity analysis. (**a, b**) Serial dilutions of in vitro–transcribed ORF1ab (**a**) and N (**b**) for LoD determination. After amplification at 42°C for 20 minutes, 10 μL of each reaction was transferred to 40 μL of the Cas12a mixture for cleavage assays. Bar graphs represent fluorescent signal obtained at 10 minutes for the Cas12a reaction. The data are presented as the means ± SD (*n* = 15). Unpaired 2-tailed *t* test was used to analyze the difference from NTC. ***P < 0.001. (**c**) The locations of primers and crRNAs of ORF1ab (upper) and N (lower) from 4 SARS-CoV-2 strains and 4 other human coronaviruses. Sequences of different types of coronavirus were obtained from GenBank and aligned by the MEGA program. Positions with complete alignment to the primer or crRNA are labeled in yellow. (**d, e**) Specificity assessment of the RT-RPA assay for ORF1ab gene (**d**) and N gene (**e**) of SARS-CoV-2. Only the RNA from SARS-CoV-2 produced signals, whereas RNAs from other pathogens and the negative control did not produce any detected signals. Four human coronaviruses (SARS, HKU1, MERS, and OC43) and 11 other respiratory pathogens including avian influenza A viruses (H7N9 and H5N1), human influenza A viruses (H1N1 [swine flu] and H3N2 subtypes), influenza B viruses (Yamagata and Victoria lineages), adenovirus (ADV3, 7), human parainfluenza virus (PIV2), *Staphylococcus aureus*, and *Streptococcus pneumonia* were evaluated. Numerical source data underlying this figure can be found in S1 Data. LoD, Limit of Detection; MERS, Middle East Respiratory Syndrome; NTC, no-template control; RPA, recombinase polymerase amplification; RT, room temperature; SARS, Severe Acute Respiratory Syndrome; SARS-CoV-2, Severe Acute Respiratory Syndrome Coronavirus 2; SD, standard deviation.

To determine the analytical sensitivity of this system, the targets were serially diluted to 1, 10, and 100 copies/μL. Then, 1 μL of RNA from each dilution was used as template, and 3 independent replicates each with 5 technical replicates were performed. The CRISPR-Cas12a assays for ORF1ab and N gene were both capable of detecting their corresponding targets at all assayed dilutions and did not show any signals in the no-template control (NTC, Fig 3). These results illustrate our developed CRISPR-Cas12a assay has a single-copy sensitivity.

We then aligned the primers with other coronavirus genomes (Human Coronavirus OC43 [HCoV-OC43], Human Coronavirus HKU1 [HCoV-HKU1], Severe Acute Respiratory Syndrome [SARS], and Middle East Respiratory Syndrome Coronavirus [MERS-CoV]) using MEGA 7 and found the sequences were not similar to most viral sequences (Fig 3C). Moreover, the crRNAs targeting the ORF1ab and N genes of SARS-CoV-2 have several mismatches to other coronavirus, suggesting high specificity of the assay to targets since crRNAs have been shown to be sensitive to single-nucleotide mutations [15]. We further experimentally evaluated the specificity of our assay to 4 human coronavirus strains and 11 respiratory pathogens. As shown in Fig 3D, cross-reactivity was not observed with SARS-CoV-2 and the tested respiratory pathogens. These results together demonstrate that the established system is highly specific to SARS-CoV-2. This, however, may be a double-edged sword, because false negative may occur when analyzing clinical samples in which mutations commonly existed [22].

**Fig 4:**
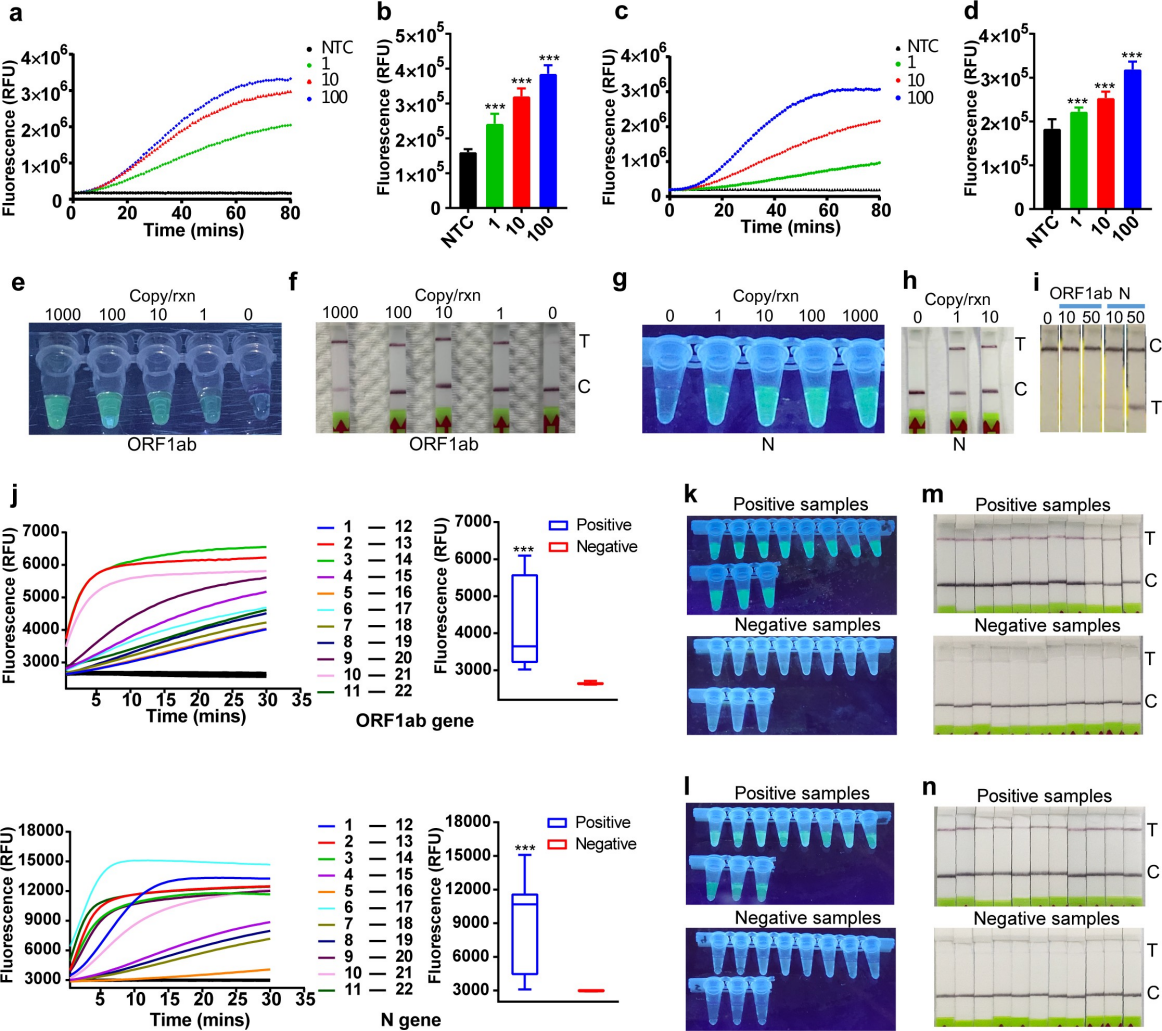
Validation of the system with pseudovirus and clinical samples. **(a–d)** The LoD was determined using the lysed pseudovirus for ORF1ab (**a, b**) and N (**c, d**). Fluorescent signal was obtained at 10 minutes for the Cas12a reaction (**b, d**). Fifteen replicates were conducted for each test. The data are presented as the means ± SD. Unpaired 2-tailed *t* test was used to analyze the difference from NTC. ***P < 0.001. (**e, g)** The signals of ORF1ab (**e**) and N (**g**) were detected with a gel imaging system after 20 minutes of Cas12a cleavage at the indicated viral load of pseudovirus, and the signals were visible with the naked eye; rnx, per reaction. (**f, h**) Lateral flow detection of ORF1ab (**f**) and N (**h**) genes at the indicated viral load. T, test line; C, control line; rnx, per reaction. (**i**) RPA-only detection of ORF1ab and N gene. RT-RPA reaction was performed at 42°C for 12 minutes, after which the product was diluted 1:5 in HybriDetect Assay Buffer, and the strips were inserted and incubated for 2 minutes at RT. Then, the strips were removed and imaged using a smartphone camera. T, test line; C, control line. (**j)** Real-time (left panel) and end point (right panel) fluorescence detection using primers specific to ORF1ab and N gene of SARS-CoV-2 in clinical samples. Eleven PCR–positive and PCR–negative samples each with 2 μl of the RNA input were used for the evaluation. 1–11, PCR–positive samples; 12–22, PCR–negative samples. Box-and-whisker plots represent fluorescent values at 20 minutes for the Cas12a reaction. The lower and upper borders of the box represent first and third quartile of the values, respectively. The line within the box shows the median, and the whiskers falling outside the box are plotted as the minimum and maximum of the data. Unpaired 2-tailed *t* test was used to analyze the difference from negatives (*n* = 11); ***P < 0.001. (**k, l**) Visual detection for ORF1ab (**k**) and N (**l**) genes of SARS-CoV-2 in clinical samples. Eleven PCR–positive and PCR–negative samples were used for the evaluation. The signals were obtained with a gel imaging machine at 20 minutes for the Cas12a reaction. (**m, n**) Eleven PCR–positive and PCR–negative clinical samples were detected for ORF1ab (**m**) and N (**n**) genes of SARS-CoV-2 using lateral flow strips. RT-RPA reaction was performed at 42°C for 20 minutes and followed by 20 minutes of Cas12a cleavage, and then the product was diluted and overflowed on a strip for 2 minutes. T, test line; C, control line. Numerical source data underlying this figure can be found in S1 Data. Cas, CRISPR associated proteins; LoD, Limit of Detection; NTC, no-template control; PCR, polymerase chain reaction; RPA, recombinase polymerase amplification; RT, room temperature; SARS-CoV-2, Severe Acute Respiratory Syndrome Coronavirus 2; SD, standard deviation.

Next, to mimic clinical situations, we spiked pseudovirus containing fragments of ORF1ab and N genes into samples from healthy volunteers. As compared to a live virus, pseudoviruses, which can be produced naturally during the infection or artificially by researchers in laboratories, contain nucleic acid sequences of interest but do not have any of the components for infectious viruses. We tested various protocols for sample lysis and found that the one that used Triton X-100, Tween-20, guanidine hydrochloride, and a heating step yielded the strongest signal (S1a Fig). Nevertheless, the signal was significantly reduced when the heating step was abolished (S1b Fig), implying incomplete sample lysis without heating. This protocol allowed the sample processing to be finished in 5 minutes, and the unextracted lysate could be directly served as templates for RT-RPA amplification. As shown in Fig 4A–4D, our assay was able to detect a single copy of pseudovirus in a reaction.

Visual detection is crucial for nucleic acid diagnostics, especially in situations when instruments are not available. In this study, we used a reporter with a dye label (FAM) on the 5′ end and a fluorescent quencher (BHQ1) on the 3′ end. After the Cas12a cleavage assay, we observed bright green signals by the naked eye under a gel imager when the targets were present (Fig 4E and 4G). With this method, a detection as sensitive as 1 copy per reaction was realized, which is comparable with the method using a fluorescence plate reader. We developed another reporter labeled with a FAM molecule on the 5′ end and a biotin on the 3′ end. When coupled with a lateral flow strip, the destruction of the reporter is visible by the naked eye [10,21]. This method exhibited an analytical sensitivity of 1 copy/μL for the template (Fig 4F and 4H).

We next compared the sensitivity of the CRISPR-Cas12a-based method with the RPA-only method, the latter of which appeared not to be as sensitive as the former with observed Limit of Detection (LoD) values of 50 copies/μL and 10 copies/μL for the ORF1ab and N genes, respectively, in lateral flow assay (Fig 4I). In fluorescent detection assay, the RPA-only detection also showed an inferior sensitivity than Cas12a-based assay with LoD of 1,000 and 100 copies for ORF1ab and N genes, respectively, in a given reaction (S2 Fig). This observation may ascribe to the signal amplification effect induced by the collateral activity of Cas12a [23]. Therefore, it would be reasonable to speculate that the CRISPR-Cas12a-based detection approach is more sensitive than the RPA-only system in general.

Finally, we evaluated our system using 22 clinical samples initially diagnosed by RT-qPCR. The results showed 100% consistency between our assay and the RT-qPCR readouts for both the negative and positive samples (Fig 4J–4N, S3 Fig). Of note, 1 sample (NO. 5) showed slow increase of fluorescence for N gene detection, which is in agreement with the quantitative polymerase chain reaction (qPCR) data, where the highest cycle threshold (CT) value was observed (S4 Fig). However, it displayed bright green under UV, as well as an obvious test band on the strip, indicating that the visual detection system is of high sensitivity. This results should be carefully interpreted due to the insufficient number of samples. A larger cohort would be helpful to better understand the sensitivity and specificity and also provide confidence that this assay is adequate to address the pandemic’s needs for scalable and accurate testing.

In summary, we report the development and initial validation of a CRISPR-Cas12-based method for detection of SARS-CoV-2 using purified RNA or rapidly lysed samples. This 2-step method requires opening of the tube after RPA reaction, which may generate aerosol and cause false-positive results. To avoid cross contamination, the tube opening when preparing Cas12a detection solution should be strictly performed in a separate area. Another smart strategy was to add the Cas enzyme on the inner wall of the reaction tube, and an extra centrifugation step was followed to initiate the detection after RPA amplification [23]. When performing the lateral flow test, a single-use cartridge that can internally direct the Cas12a reaction toward the dipstick for readout could be used to reduce the risk of amplicon contamination [24].

Since there is still no specific medicines available for the treatment of coronavirus pneumonia, early detection and treatment are extremely vital to patients. The application of this rapid, sensitive, and portable system, to some extent, could help in the control of the Coronavirus Disease 2019 (COVID-19).

## Materials and methods

### Ethical statement

A total of 22 clinical samples (nasopharyngeal swabs), including 11 positive and 11 negative samples, were collected at Shenzhen Luohu People’s Hospital between February and May of 2020. Oral consent was obtained from all enrolled patients. The collection and detection for SARS-CoV-2 virus were approved by the Shenzhen Centers for Disease Control and Prevention (CDC). The research on rapid diagnostic technique for SARS-CoV-2 using clinical samples was approved by the ethical committee of Shenzhen Luohu People’s Hospital (NO. 2020-LHQRMYY-LL-002).

### Template RNA production

Fragments of ORF1ab and N gene of SARS-CoV-2 and other human coronaviruses were synthesized and ligated into a pUC19 vector (BIOLIGO, Shanghai, China). The fragments were PCR amplified using primers containing T7 promoter and 20 nucleotide target sequences. The amplified PCR product served as the DNA template for in vitro transcription (IVT) reactions with a commercially available kit (Thermo Fisher Scientific, New Jersey, United States of America) according to the manufacturer’s instructions. The copy number of RNA was calculated based on the concentration quantified by a spectrophotometer (Thermo Fisher Scientific) using the following equation: RNA copy number = (M × 6.022 × 10^23^)/(n × 1× 10^9^ × 330), in which M represents the amount of RNA in nanograms, n is the length of the RNA in base, and the average weight of a base is assumed to be 330 Daltons.

### Primer design and RPA reactions

RPA primers targeting the ORF1ab and N genes of SARS-CoV-2 were designed as described in the protocol of Twist-Dx (Maidenhead, United Kingdom). Primer pairs were screened using a forward primer against reverse primers, where the best reverse primer was selected and then used to screen all the forward primers. The primer pairs with the best performance were used in subsequent experiments. RT-RPA reactions were performed using a kit (Amp-Future, Weifang, China) according to the manufacturer’s protocol. Briefly, the reactions were performed in a total volume of 12.5 μL comprising RT-RPA enzymes, 1 μL of RNA input, 1× rehydration buffer, 14 mM magnesium acetate, and 0.4 μM of each primer. As with the RPA-only detection, additional 120 nM probe was supplemented into the reactions. All reactions were incubated at 42°C for 15 to 20 minutes.

### Cas12a detection reactions

The RT-RPA product (10 μL) was added to 40 μL of the CRISPR-Cas12a reaction mixture containing 100 nM crRNA (BIOLIGO), 50 nM Cas12a (NEB, Ipswich, UK), and 250 nM ssDNA reporter (BIOLIGO). Then, the reactions (50 μL in a 96-well microplate or in a PCR tube) were incubated in a fluorescence plate reader (Molecular Devices, California, USA) or a Real-Time PCR Detection System (Bio-Rad, Watford, UK) for up to 120 minutes at 37°C with fluorescent signals collected every 30 seconds (ssDNA FQ substrates = λex: 485 nm; λem: 535 nm). With respect to visual detection, all CRISPR reactions were incubated at 37°C for 15 to 20 minutes.

### Analytical specificity of the RT-RPA assay

The assay specificity was evaluated by testing RNAs from human coronaviruses (SARS, HKU1, MERS, and OC43), avian influenza A viruses (H7N9 and H5N1), human influenza A viruses (H1N1 [swine flu] and H3N2 subtypes), influenza B viruses (Yamagata and Victoria lineages), adenovirus (ADV3, 7), human parainfluenza virus (PIV2), *Staphylococcus aureus*, and *Streptococcus pneumonia*. The RNAs of human coronaviruses were generated by IVT, and the reference RNAs of other respiratory pathogens was purchased from the National Institutes for Food and Drug Control (Beijing, China) and dissolved using 500 μL of RNase-free water according to the manufacturer’s instructions.

### Lateral flow detection reactions

Lateral flow detection was performed with commercial strips (TwistDx, Cambridge, UK). The Cas12a reactions were diluted 1:5 in HybriDetect Assay Buffer, after which the strips were inserted and incubated for 2 minutes at room temperature. Then, the strips were removed and imaged using a smartphone camera.

### RNA release from pseudovirus

The samples from negative volunteers were collected using a throat swab and lysed in buffers with distinguished recipes (1, 100 mM TCEP + 0.5% Triton X-100; 2, 0.5% Triton X-100 + 0.5% Tween-20; 3, 0.5% Triton X-100 + 0.5% Tween-20 + 1 mM EDTA; 4, 800 mM guanidine hydrochloride + 0.5% Triton X-100 + 1 mM EDTA; 5, 800 mM guanidine hydrochloride + 0.5% Triton X-100 + 0.5% Tween-20). The pseudovirus purchased from Yeasen (Shanghai, China) in an original concentration of 5 × 10^6^ copies/mL was spiked into the lysis buffer, which was subsequently heated at 80°C for 5 minutes using a dry heat block. The lysates were then serially diluted to the concentration of 1 copy/μL, 10 copies/μL, 100 copies/μL, and 1,000 copies/μL.

### Human clinical sample collection and preparation

Oropharyngeal swab samples from patients infected with SARS-CoV-2 were immediately placed into sterile tubes containing 3 mL of viral transport media (VTM, Health Gene Technologies, Ningbo, China). The collected samples were deactivated by heating at 56°C for 30 minutes in a biosafety level 2 (BSL 2) medical laboratory of Shenzhen Luohu People’s Hospital in China. RNA was purified with an RNA extraction kit (Health Biomed, Shanghai, China) on a Smart LabAssist-32 platform (Taiwan Advanced Nanotech, Taoyuan, China). To avoid the interference of TE buffer to RT-RPA reaction, RNA was eluted with RNase- and DNase-free water.

### qPCR assay for SARS-CoV-2 testing

The viral nucleic acids were detected with a SARS-CoV-2 test kit (Yaneng Biotech, Shenzhen, China) according to manufacturer’s instruction. The assay was performed using a SLAN96P Instrument (Hongshi, Shanghai, China), with the following program: reverse transcription at 50°C for 15 minutes, pre-denaturation at 95°C for 10 minutes, and followed by 45 cycles of denaturation at 95°C for 10 seconds and annealing and extension at 55°C for 40 seconds.

All the primers and crRNAs used in this study are listed in S1 and S2 Tables. The general illustration of our assay is shown in Fig 1, which consists of a sample processing step at 80°C for 5 minutes, an RT-RPA reaction at 42°C for 15 to 20 minutes, and a Cas12 detection performed at 37°C for 15 to 20 minutes. This assay can be run in approximately 35 to 50 minutes and be visualized under a UV light imager (Jiapeng Tech., Shanghai, China) or a lateral flow strip.

## Supporting information

S1 Fig Optimization of the rapid sample lysis protocol. (**a**) Representative plot of fluorescence intensity versus time for lysis buffer screening using pseudovirus containing fragments of N gene of SARS-CoV-2 (left panel). Fluorescent signal was obtained at 10 minutes for the Cas12a reaction (right panel). Error bars represent the mean ± SD, where *n* = 3 replicates. The pseudovirus was lysed at 80°C for 5 minutes. Conditions: 1, 100 mM TCEP + 0.5% Triton X-100; 2, 0.5% Triton X-100 + 0.5% Tween-20; 3, 0.5% Triton X-100 + 0.5% Tween-20 + 1 mM EDTA; 4, 800 mM guanidine hydrochloride + 0.5% Triton X-100 + 1 mM EDTA; 5, 800 mM guanidine hydrochloride + 0.5% Triton X-100 + 0.5% Tween-20. (**b**) Real-time (left panel) and end point (right panel) fluorescence detection for N gene of SARS-CoV-2. Viruses were lysed at 80°C (5-heated) or room temperature (5-RT) for 5 minutes in a buffer containing 800 mM guanidine hydrochloride, 0.5% Triton X-100, and 0.5% Tween-20. Error bars represent the mean ± SD, where *n* = 3 independent repeats each with 3 technical replicates. Numerical source data underlying this figure can be found in S1 Data. Cas, CRISPR associated proteins; SARS-CoV-2, Severe Acute Respiratory Syndrome Coronavirus 2; SD, standard deviation.
(TIF)

S2 Fig RPA-only detection of ORF1ab and N genes of SARS-CoV-2. Fluorescent kinetic curves on ORF1ab (left) and N (right) genes of SARS-CoV-2. The reactions with the indicated copy number of RNA were incubated at 37°C for 20 minutes with fluorescence measured every 30 seconds. Numerical source data underlying this figure can be found in S1 Data. RPA, recombinase polymerase amplification; SARS-CoV-2, Severe Acute Respiratory Syndrome Coronavirus 2.
(TIF)

S3 Fig Guidance for interpretation of the visual detection results [(a) lateral flow detection; (b) UV light detection] of SARS-CoV-2. SARS-CoV-2, Severe Acute Respiratory Syndrome Coronavirus 2; UV, ultraviolet.
(TIF)

S4 Fig qPCR validation of 11 COVID-19–infected samples and 11 patient samples for other viral respiratory infections. CT values using the qPCR assay for detection of the ORF1ab and N gene of SARS-CoV-2. 1–11, PCR–positive samples; 12–22, PCR–negative samples. All undetected CT values in negative samples were presented as 40. Numerical source data underlying this figure can be found in S1 Data. COVID-19, Coronavirus Disease 2019; CT, cycle threshold; PCR, polymerase chain reaction; qPCR, quantitative polymerase chain reaction; SARS-CoV-2, Severe Acute Respiratory Syndrome Coronavirus 2.
(TIF)

S1 Table Primers, crRNAs, and reporters used for CRISPR-Cas12a-based SARS-CoV-2 detection. Cas, CRISPR associated proteins; CRISPR, clustered regularly interspaced short palindromic repeats; SARS-CoV-2, Severe Acute Respiratory Syndrome Coronavirus 2.
(DOCX)

S2 Table Primers and probes used for RPA-only detection. RPA, recombinase polymerase amplification.
(DOCX)

S1 Data Data underlying Figs 2, 3A, 3B, 3D, 3E, 4A–4D, 4J and S1A and S1B, S2 and S4 Figs. (XLSX)

## Abbreviations

BSL 2: biosafety level 2
Cas: CRISPR associated proteins
CDC: Centers for Disease Control and Prevention
COVID-19: Coronavirus Disease 2019
CRISPR: clustered regularly interspaced short palindromic repeats
CT: cycle threshold
HCoV-HKU1: Human Coronavirus HKU1
HCoV-OC43: Human Coronavirus OC43
IVT: in vitro transcription
LoD: Limit of Detection
MERS: Middle East Respiratory Syndrome
MERS-CoV: Middle East Respiratory Syndrome Coronavirus
NTC: no-template control
qPCR: quantitative polymerase chain reaction
RPA: recombinase polymerase amplification
RT-qPCR: reverse transcription polymerase chain reaction
SARS: Severe Acute Respiratory Syndrome
SARS-CoV-2: Severe Acute Respiratory Syndrome Coronavirus 2
ssDNA: single-stranded DNA
VTM: viral transport media
