# Supplementary figures and images for: Rapid detection of SARS-CoV-2 with CRISPR-Cas12a
Source: PLoS Biol. 2020 Dec 15;18(12):e3000978. doi: 10.1371/journal.pbio.3000978 (PMC7737895; doi:10.1371/journal.pbio.3000978)

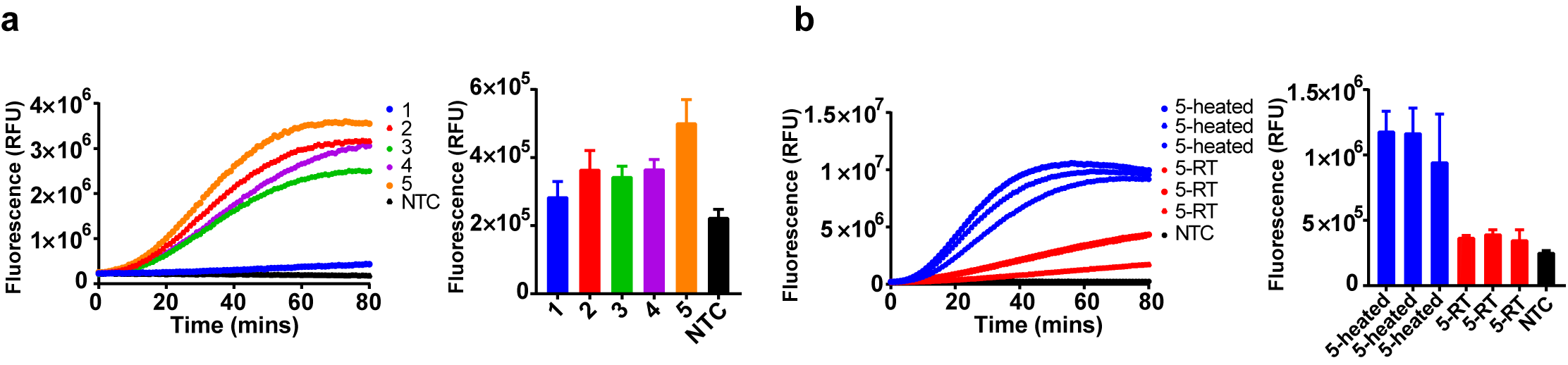

Supplement: S1 Fig — (a) Representative plot of fluorescence intensity versus time for lysis buffer screening using pseudovirus containing fragments of N gene of SARS-CoV-2 (left panel). Fluorescent signal was obtained at 10 minutes for the Cas12a reaction (right panel). Error bars represent the mean ± SD, where n = 3 replicates. The pseudovirus was lysed at 80°C for 5 minutes. Conditions: 1, 100 mM TCEP + 0.5% Triton X-100; 2, 0.5% Triton X-100 + 0.5% Tween-20; 3, 0.5% Triton X-100 + 0.5% Tween-20 + 1 mM EDTA; 4, 800 mM guanidine hydrochloride + 0.5% Triton X-100 + 1 mM EDTA; 5, 800 mM guanidine hydrochloride + 0.5% Triton X-100 + 0.5% Tween-20. (b) Real-time (left panel) and end point (right panel) fluorescence detection for N gene of SARS-CoV-2. Viruses were lysed at 80°C (5-heated) or room temperature (5-RT) for 5 minutes in a buffer containing 800 mM guanidine hydrochloride, 0.5% Triton X-100, and 0.5% Tween-20. Error bars represent the mean ± SD, where n = 3 independent repeats each with 3 technical replicates. Numerical source data underlying this figure can be found in S1 Data. Cas, CRISPR associated proteins; SARS-CoV-2, Severe Acute Respiratory Syndrome Coronavirus 2; SD, standard deviation. (TIF) [file pbio.3000978.s001.tif]

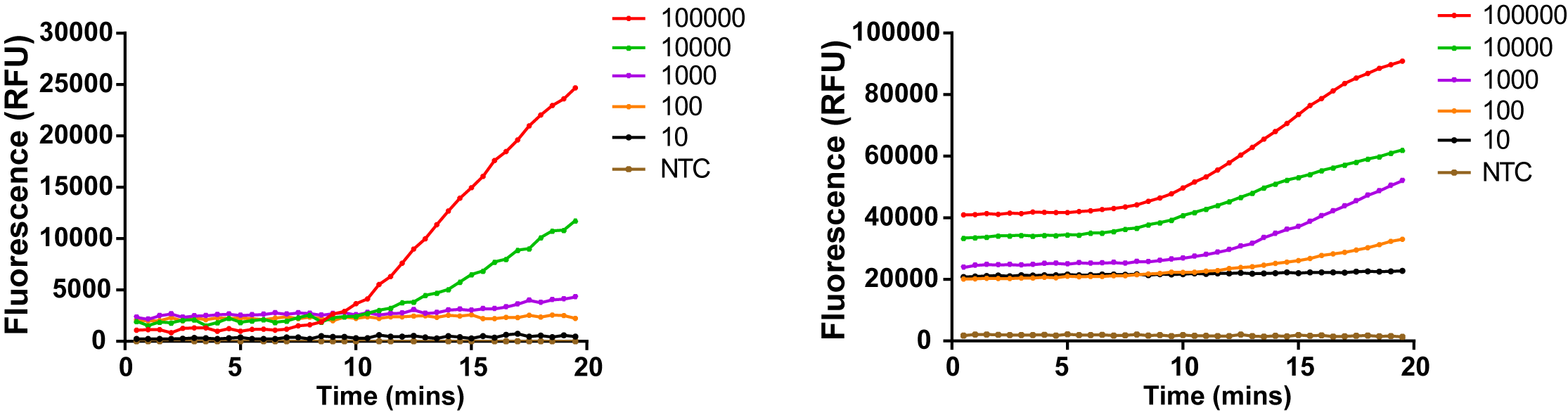

Supplement: S2 Fig — Fluorescent kinetic curves on ORF1ab (left) and N (right) genes of SARS-CoV-2. The reactions with the indicated copy number of RNA were incubated at 37°C for 20 minutes with fluorescence measured every 30 seconds. Numerical source data underlying this figure can be found in S1 Data. RPA, recombinase polymerase amplification; SARS-CoV-2, Severe Acute Respiratory Syndrome Coronavirus 2. (TIF) [file pbio.3000978.s002.tif]

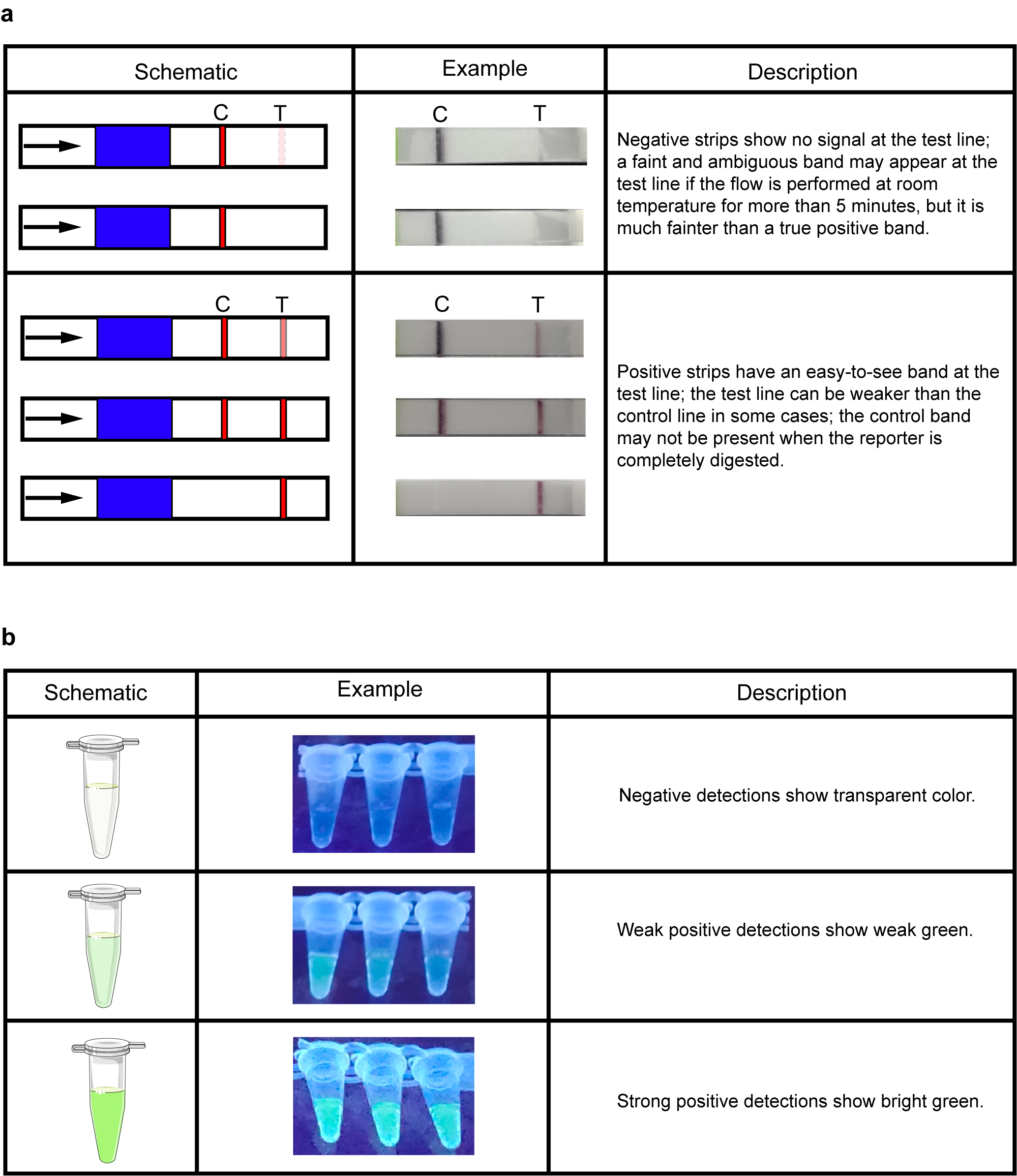

Supplement: S3 Fig — Guidance for interpretation of the visual detection results [(a) lateral flow detection; (b) UV light detection] of SARS-CoV-2. SARS-CoV-2, Severe Acute Respiratory Syndrome Coronavirus 2; UV, ultraviolet. (TIF) [file pbio.3000978.s003.tif]

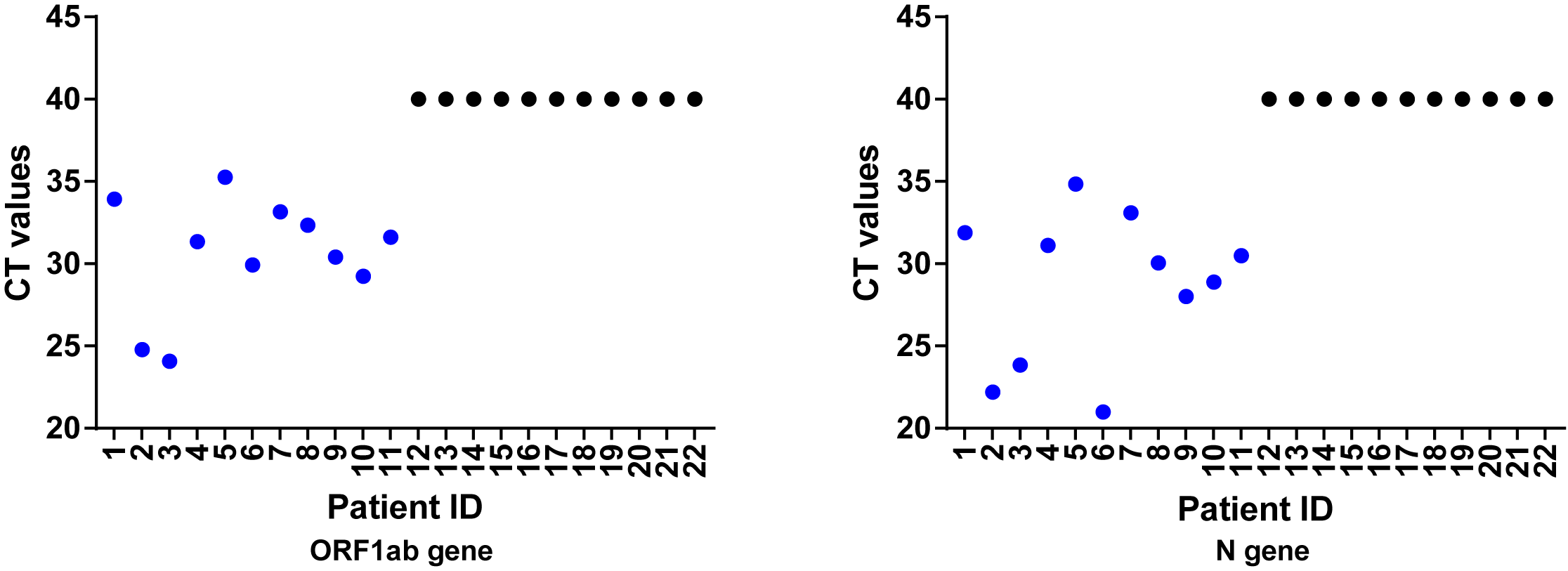

Supplement: S4 Fig — CT values using the qPCR assay for detection of the ORF1ab and N gene of SARS-CoV-2. 1–11, PCR–positive samples; 12–22, PCR–negative samples. All undetected CT values in negative samples were presented as 40. Numerical source data underlying this figure can be found in S1 Data. COVID-19, Coronavirus Disease 2019; CT, cycle threshold; PCR, polymerase chain reaction; qPCR, quantitative polymerase chain reaction; SARS-CoV-2, Severe Acute Respiratory Syndrome Coronavirus 2. (TIF) [file pbio.3000978.s004.tif]
