# Supplementary material for: Rapid detection of SARS-CoV-2 with CRISPR-Cas12a
Source: PLoS Biol. 2020 Dec 15;18(12):e3000978. doi: 10.1371/journal.pbio.3000978 (PMC7737895; doi:10.1371/journal.pbio.3000978)
Supplement: S1 Table — Cas, CRISPR associated proteins; CRISPR, clustered regularly interspaced short palindromic repeats; SARS-CoV-2, Severe Acute Respiratory Syndrome Coronavirus 2. (DOCX) [file pbio.3000978.s005.docx]

S1 Table. Primers, crRNAs and reporters used for CRISPR-Cas12a-based SARS-CoV-2 detection.

| **Oligo name** | **Sequence(5'-3')** |
| --- | --- |
| ORF1ab-F1 | GATTGCTGCAGTCATAACAAGAGAAGTGGGTT |
| ORF1ab-F2 | TTGCTGCAGTCATAACAAGAGAAGTGGGTTTT |
| ORF1ab-F3 | CTGCAGTCATAACAAGAGAAGTGGGTTTTGTC |
| ORF1ab-F4 | AGTCATAACAAGAGAAGTGGGTTTTGTCGTGCC |
| ORF1ab-F5 | TTGCCTGGCACGATATTACGCACAACTAATGGT |
| ORF1ab-R1 | CAAGCTGATGTTGCAAAGTCAGTGTACTCTAT |
| ORF1ab-R2 | GCTGATGTTGCAAAGTCAGTGTACTCTATAAG |
| ORF1ab-R3 | TGATGTTGCAAAGTCAGTGTACTCTATAAGTTT |
| ORF1ab-R4 | TGTTGCAAAGTCAGTGTACTCTATAAGTTTTGA |
| ORF1ab-R5 | TTGCAAAGTCAGTGTACTCTATAAGTTTTGATG |
| N-F1 | CACATTGGCACCCGCAATCCTGCTAACAAT |
| N-F2 | TTGGCACCCGCAATCCTGCTAACAATGCTGCA |
| N-F3 | ACCCGCAATCCTGCTAACAATGCTGCAATCGT |
| N-F4 | CTTCCTCAAGGAACAACATTGCCAAAAGGCT |
| N-R1 | CAGGAGAAGTTCCCCTACTGCTGCCTGGAGTT |
| N-R2 | TCTAGCAGGAGAAGTTCCCCTACTGCTGCCTGG |
| N-R3 | GCCATTCTAGCAGGAGAAGTTCCCCTACTGCTG |
| crRNA-ORF1ab | UAAUUUCUACUAAGUGUAGAUgugcaguugguaacaucuguuac |
| crRNA-nCOV-N | UAAUUUCUACUAAGUGUAGAUuugaacuguugcgacuacgugau |
| ssDNA FQ reporter | FAM-TTATT-BHQ1 (For fluorescence assay) |
| ssDNA FB reporter | FAM-TTATT-Biotin (For lateral flow assay) |
