# Supplementary material for: Rapid detection of SARS-CoV-2 with CRISPR-Cas12a
Source: PLoS Biol. 2020 Dec 15;18(12):e3000978. doi: 10.1371/journal.pbio.3000978 (PMC7737895; doi:10.1371/journal.pbio.3000978)
Supplement: S2 Table — RPA, recombinase polymerase amplification. (DOCX) [file pbio.3000978.s006.docx]

S2 **Ta**ble. Primers and probes used for RPA-only detection

| ORF1ab-P1 | FAM-CATTTCTTACCTAGAGTTTTTAGTGCAGTTHGTAACATCTGTTACA-C3 spacer | For lateral flow assay  (H, tetrahydrofuran) |
| --- | --- | --- |
| ORF1ab-F5 | TTGCCTGGCACGATATTACGCACAACTAATGGT |  |
| ORF1ab-R6 | Biotin-GCTGATGTTGCAAAGTCAGTGTACTCTATAAG |  |
| N-P1 | FAM-GGCAATGGCGGTGATGCTGCTCTTGCTTTGHTGCTGCTTGACAGAT-C3 spacer | For lateral flow assay  (H, tetrahydrofuran) |
| N-F5 | CAGGCAGCAGTAGGGGAACTTCTCCTGCTAGAAT |  |
| N-R4 | Biotin-GTTGGCCTTTACCAGACATTTTGCTCTCAAGCTG |  |
| ORF1ab-F2 | TTGCTGCAGTCATAACAAGAGAAGTGGGTTTT | For fluorescence assay  (H, tetrahydrofuran) |
| ORF1ab-R2 | GCTGATGTTGCAAAGTCAGTGTACTCTATAAG |  |
| ORF1ab-P2 | TTACGCACAACTAATGGTGACTTTTTGCATTTCT dT-FAM A H C dT-BHQ1 AGAGTTTTTAGTGC-C3 spacer |  |
| N-P2 | CGCAGAAGGGAGCAGAGGCGGCAGTCAAGCCTCTTC dT-HEX C H T dT-BHQ1 CCTCATCACGTAGT-C3 spacer | For fluorescence assay  (H, tetrahydrofuran) |
| N-F4 | CTTCCTCAAGGAACAACATTGCCAAAAGGCT |  |
| N-R5 | GCCATTGCCAGCCATTCTAGCAGGAGAAGTTCC |  |
